# Serum levels of interleukins and S100A8/A9 correlate with clinical severity in patients with dermatomyositis-associated interstitial lung disease

**DOI:** 10.1186/s12890-020-01226-3

**Published:** 2020-07-17

**Authors:** Yueyan Lou, Yu Zheng, Bijun Fan, Liyan Zhang, Feng Zhu, Xiaodong Wang, Zhiwei Chen, Xiaoming Tan, Qing Wei

**Affiliations:** 1 Department of Pulmonology, Renji Hospital South Campus, Shanghai Jiaotong University School of Medicine, Shanghai, People’s Republic of China; 2 Department of Rheumatology, Renji Hospital South Campus, Shanghai Jiaotong University School of Medicine, Shanghai, People’s Republic of China; 3 Department of Laboratory Medicine, Renji Hospital South Campus, Shanghai Jiaotong University School of Medicine, Shanghai, People’s Republic of China

**Keywords:** Dermatomyositis, Interstitial lung disease, S100A8/A9, Interleukin

## Abstract

**Background:**

Dermatomyositis (DM) is a systemic autoimmune inflammatory disorder that affects primarily skin, muscle and lung, frequently associated with interstitial lung disease (ILD). The objective of this study is to investigate the association between serum cytokines and clinical severity in patients with DM-ILD.

**Methods:**

Serum samples of 30 healthy controls, 14 DM patients without ILD and 40 DM patients with ILD were collected. Serum S100A8/A9 levels were analyzed by enzyme-linked immunosorbent assay (ELISA) and levels of interleukins were measured by cytometric beads array (CBA). Then we performed multivariate logistic regression analysis to determine factors independently associated with ILD development.

**Results:**

Serum IL-4, IL-6 and S100A8/A9 levels were significantly higher in DM patients with ILD than those in healthy controls (*p* = 0.0013, 0.0017 and < 0.0001, respectively). Serum IL-10 level of patients was dramatically lower than that in controls (*p* = 0.0001). In DM patients, the levels were significantly higher in patients with A/SIP than in those with CIP (*p* = 0.0046, 0.0339 and 0.0133) or without ILD (*p* = 0.0165, 0.0370 and < 0.0001). IL-4 (*r* = 0.1171, *p* = 0.0040), IL-6 (*r* = 0.1174, *p* = 0.0040) and IL-10 (*r* = − 0.1829, *p* = 0.0003) were significantly correlated with S100A8/A9 in DM-ILD patients. S100A8/A9 was significantly correlated with high-resolution computed tomography (HRCT) (*r* = 0.1642, *p* = 0.0157) and lung function (DLCO%: *r* = − 0.2066, *p* = 0.0061, FVC%: *r* = − 0.2156, *p* = 0.0050). Moreover, logistic regression analysis revealed that S100A8/A9 levels were independently associated with ILD development in DM patients (*p* = 0.004).

**Conclusions:**

Serum level of S100A8/A9 may be a valuable predictor for assessing the clinical severity of DM-ILD patients. Serum IL-4, IL-6 and IL-10 levels were highly correlated with S100A8/A9, so these cytokines may play a synergistic effect on the progression of DM-ILD.

## Background

Dermatomyositis (DM) is a kind of idiopathic inflammatory myopathy (IIM), which mainly involves the inflammation of skeletal muscle and skin, and can cause muscle weakness and rash [1]. Interstitial lung disease (ILD) is considered a common systemic complication of DM [2]. DM associated with ILD is one of the major prognostic determinants, causing increased morbidity and mortality [3–5]. ILD is one of the life-threatening complications of clinically amyopathic dermatomyositis (CADM). ILD can be divided into acute/subacute interstitial pneumonia (A/SIP) and chronic interstitial pneumonia (CIP). CADM patients with A/SIP showed a rapid progressive pattern and usually resistant to intensive therapy. Therefore, early diagnosis of ILD, especially A/SIP as a complication of CADM is critical.

Autoimmune abnormalities are commonly found in inflammatory disorders and are considered to participate in the pathogenesis of DM [6]. The inflammatory infiltrates are mainly composed of T lymphocytes and macrophages in muscle tissues of patients with DM. Numerous studies to date have indicated that CD4^+^ T-helper (Th) cells may play a critical role in the pathogenesis of inflammatory myopathies [7–9]. CD4^+^ T lymphocytes are classified into type 1 (Th1) and type 2 (Th2) according to the cytokines produced. Interferon (IFN)-γ in Th1 and interleukin (IL)-4 in Th2 have been both measured in muscle biopsy specimens of patients with DM, suggesting the involvement of different Th subtypes in the disease [10].

Pro-inflammatory cytokines such as IL-6 and TNF-α were amplified by endogenous factors such as S100 protein) [11, 12]. These proteins are thought to be one of the “damage related molecular models” by recruiting inflammatory cells to induce inflammation [13]. S100A8 and S100A9 belong to S100 family. They are calcium binding proteins and form heterodimers. They play an important role in leukocyte trafficking and contribute to inflammatory responses [14]. S100A8/A9 is produced by infiltration of neutrophils and monocytes, rather than static macrophages or lymphocytes in the inflammatory state [15, 16]. S100A8/A9 is an endogenous ligand of Toll like receptor 4 (TLR-4), which is related to sepsis and endotoxemia and plays an important role in innate immunity [16, 17].

The S100A8/A9 heterodimer is also referred to as calprotectin or MRP8/14. It is abundantly expressed in neutrophils and monocytes and induces proinflammatory cytokines and chemokines through TLR2/TLR4-mediated signaling pathways [18]. Therefore, S100A8/A9 has been explored as a possible biomarker of disease severity in several autoimmune diseases, including juvenile dermatomyositis [19], systemic sclerosis [20] and rheumatoid arthritis [21].

The aim of this study was to investigate whether elevated serum levels of S100A8/A9 are associated with ILD development in DM patients. Furthermore, we assessed its potency as an independent biomarker to predict disease activity and to aid broad implementation into clinical practice.

## Methods

### Participants

The present study included 30 healthy controls, 14 DM patients without ILD and 40 DM patients with ILD. The patients were diagnosed as DM according to the classification criteria proposed by Bohan and Peter [22] [23]. Diagnosis of interstitial lung disease based on clinical symptoms and high-resolution computed tomography (HRCT), with or without lung biopsy findings. The diagnosis was based on the International Consensus Statement on Idiopathic Pulmonary Fibrosis issued by the American Thoracic Society and the European Respiratory Society [24]. Patients with ILD can be divided into two subsets: acute/subacute interstitial pneumonitis (A/SIP) (A rapidly progressive ILD leading to respiratory function failure within 1 or 3 months) and chronic interstitial pneumonitis (CIP) (slowly progressive presentation with gradual deterioration longer than 3 months).

They were admitted to the department of Respiration and Rheumatology at our hospital from January 2017 to December 2018. Clinical and laboratory data were collected retrospectively upon admission. Exclusion criteria were immunotherapy and hormone therapy for more than 2 weeks or longer after rash, myopathy, and ILD symptoms. This study was approved by the Institutional Review Board of RenJi Hospital (2016075). All participants included consent the study orally.

### Measurement of cytokines

Serum cytokine levels were detected by the BD™ cytometric bead array (CBA) assay (BD Biosciences, San Diego, CA, U.S.A.). The Human Th1/Th2/Th17 Cytokine Kits were used to detect the serum concentrations of IL-2, IL-4, IL-6, IL-10, tumor necrosis factor-α (TNF-α), IFN-γ and IL-17A. Human MRP8/14 (Calprotectin) ELISA Kit (Biolegend, San Diego, CA, U.S.A.) was used to measure serum S100A8/A9 level.

### Lung high-resolution CT scoring and pulmonary function evaluation

HRCT score was assessed by two pulmonary radiologists in a double-blind manner [25]. Each patient was tested for lung function test, percentage of predicted forced vital capacity (FVC%) and single-breath diffusing capacity of the lung for carbon monoxide (DLCO-SB) by an experienced technician on the Jaeger platform. (CareFusion, BD Biosciences).

### Statistical analysis

Multiple unpaired t-tests were employed for comparison of serum cytokine levels between patients and healthy controls. Relationships between cytokines were investigated using Spearman’s correlation coefficient test. Correlations of serum cytokines with disease severity were also using Spearman’s correlation coefficient test. Univariate and multivariate logistic regression analyses were used to identify independent risk factors for ILD development. The optimal cutoff values and the area under the curve (AUC) were obtained by receiver operating characteristic (ROC) curve analysis. All the data were analyzed by SPSS 23.0 and Graphpad Prism 6.0. A *P*-value of < 0.05 was regarded as statistically significant in all statistical analysis. The following symbols * *P* < 0.05; * * *P* < 0.01; * * *P* < 0.001 were used.

## Results

### Baseline characteristic

The present study included 30 healthy controls (11 males and 19 females; mean age 45.4 ± 10.3 years, range 19 to 65), 14 DM patients without ILD (3 males and 11 females; mean age 52.6 ± 19.9 years, range 17 to 80) and 40 DM patients with ILD (13 males and 27 females; mean age 50.1 ± 13.3 years, range 22 to 78). In DM-ILD patients, there were 17 with A/SIP and 23 with CIP. Serological profiling of each patient, including C-reactive protein (CRP), erythrocyte sedimentation rate (ESR), Ferritin and creatine kinase (CK) was performed using the standard methods. The clinical characteristics were shown in Table 1.

**Table 1:** Clinical characteristic of the DM-ILD patients and healthy controls

Variables	Healthy Controls (*n* = 30)	DM without ILD (*n* = 14)	DM-ILD (*n* = 40)
Age at onset (years), mean ± SD	45.4 ± 10.3	52.6 ± 19.9	50.1 ± 13.3
Gender (female/male)	19/11	11/3	27/13
Subtype of DM (DM/CADM)		12/2	18/22
Subtype of ILD			
A/SIP in all			17
CIP in all			13
Pulmonary function tests			
HRCT score			144.5 ± 37.21
%FVC (%)		84.2 ± 14.6	67.2 ± 19.6
%DLCO (%)		52.0 ± 26.3	37.4 ± 18.5
Laboratory findings			
CRP (mg/dL)	4.1 ± 3.0	5.9 ± 5.4	13.5 ± 15.5
ESR (mm/h)	7.7 ± 6.0	20.6 ± 14.4	24.9 ± 21.5
Ferritin (ng/mL)	172.6 ± 147.5	238.0 ± 244.7	758.7 ± 1036.7
CK (U/l)	75.3 ± 29.2	100.0 ± 49.3	152.3 ± 204.7

^a^*A/SIP* Acute/subacute interstitial pneumonia, *CADM* clinically amyopathic dermatomyositis, *CIP* Chronic interstitial pneumonia, *CK* creatine kinase, *CRP* C-reactive protein, *DLCO%* % diffusing capacity of the lungs for carbon monoxide, *DM* dermatomyositis, *ESR* erythrocyte sedimentation rate, *FVC%* % forced vital capacity, *HRCT* high-resolution computed tomography, *ILD* interstitial lung disease

### Comparison of serum cytokine levels between DM patients without ILD and those with ILD

**Fig. 1:**
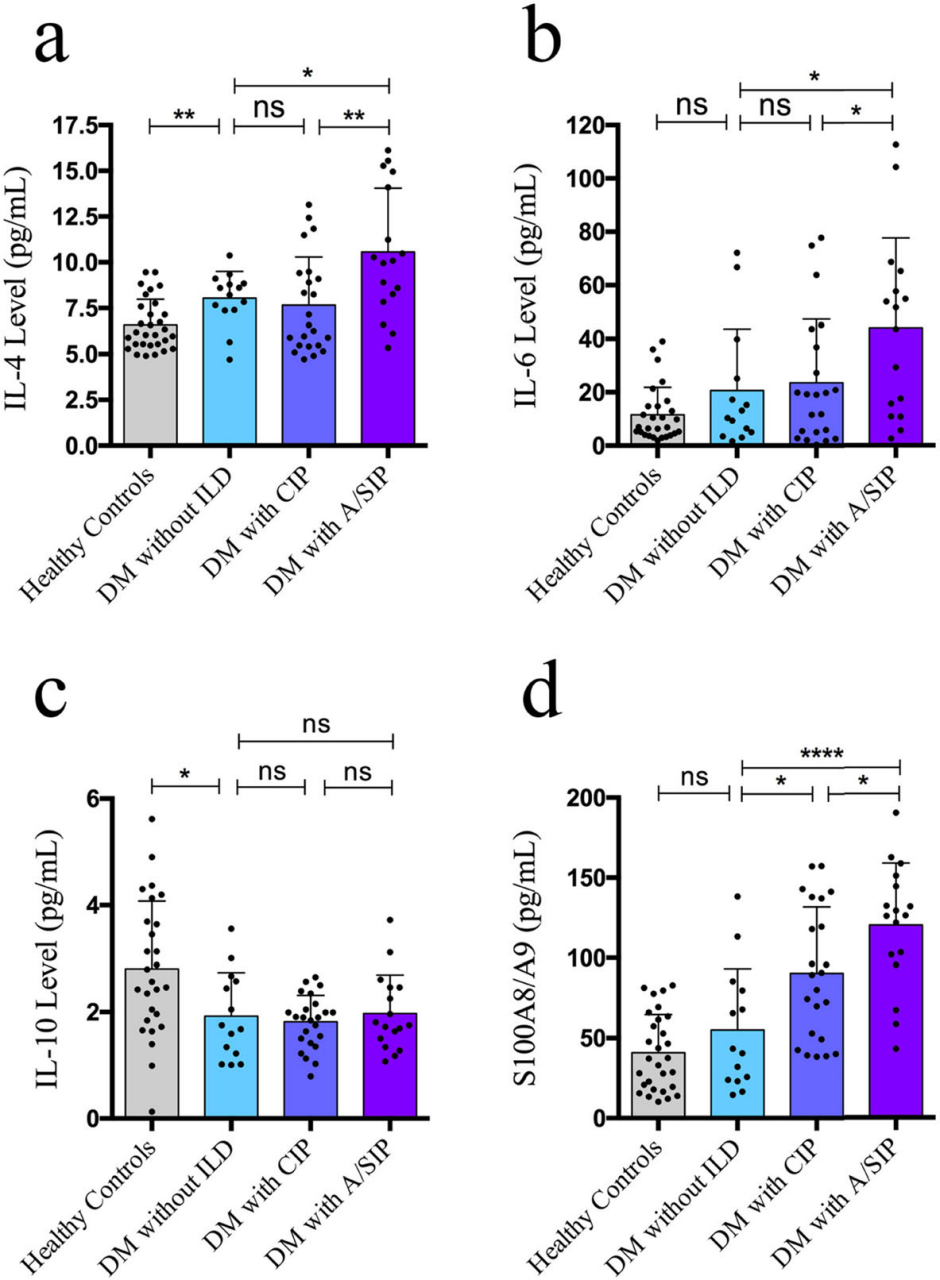
Comparison of serum cytokine levels among DM patients without ILD, those with CIP and those with A/SIP. Serum S100A8/A9 levels **a** were determined by ELISA, and IL-4 **b**, IL-6 **c** and IL-10 **d** levels were analyzed by cytometric bead array (CBA). Statistical analysis was performed using the multiple unpaired t test. **P < 0.01, ***P < 0.001

To investigate the association of levels of serum cytokines and ILD progression in DM patients, we compared their levels among DM patients with A/SIP (*n* = 17), those with CIP (*n* = 23), those without ILD (*n* = 14) and healthy controls (*n* = 30). In CBA studies, serum IL-4 (9.743 ± 0.7713 versus 6.594 ± 0.2539, *P* = 0.0013) and IL-6 (44.10 ± 8.109 versus 11.57 ± 1.937, *P* = 0.0017) levels were significantly higher in patients with DM-ILD than healthy controls. Serum IL-10 level in patients was significantly lower than controls (1.881 ± 0.09292 versus 2.809 ± 0.2445, *P* = 0.0001). In ELISA analysis, DM-ILD patients had significantly higher S100A8/A9 levels than controls (103.1 ± 6.692 versus 40.92 ± 4.430, *P* < 0.0001). No significant differences in the levels of IL-2, TNF-α, IFN-γ and IL-17A were detected between DM-ILD patients and controls (Table 2). Moreover, In DM patients, the levels of IL-4, IL-6 and S100A8/A9 were significantly higher in patients with A/SIP than in those with CIP (*p* = 0.0046, 0.0339 and 0.0133) or without ILD (*p* = 0.0165, 0.0370 and < 0.0001) (Fig. 1).

**Table 2:** Cytokine levels of the DM-ILD patients and healthy controls

	DM-ILD (*n* = 40) Mean ± SD, pg/mL	Controls (*n* = 30) Mean ± SD, pg/mL	*P* value
IL-2	4.569 ± 0.0424	4.635 ± 0.0551	0.3413
IL-4	9.743 ± 0.7713	6.594 ± 0.2539	0.0013**
IL-6	44.10 ± 8.1091	11.57 ± 1.9372	0.0017**
IL-10	1.881 ± 0.0929	2.809 ± 0.2445	0.0001***
TNF-α	2.366 ± 0.1400	2.525 ± 0.1301	0.4280
IFN-γ	3.505 ± 0.1612	3.604 ± 0.1496	0.6727
IL-17A	10.525 ± 0.1483	9.367 ± 0.0722	0.5725
S100A8/A9	103.1 ± 6.6925	40.92 ± 4.4302	< 0.0001***

**P < 0.01, ***P < 0.001

### Correlation between the levels of IL-4, IL-6, IL-10 and S100A8/A9

**Fig. 2:**
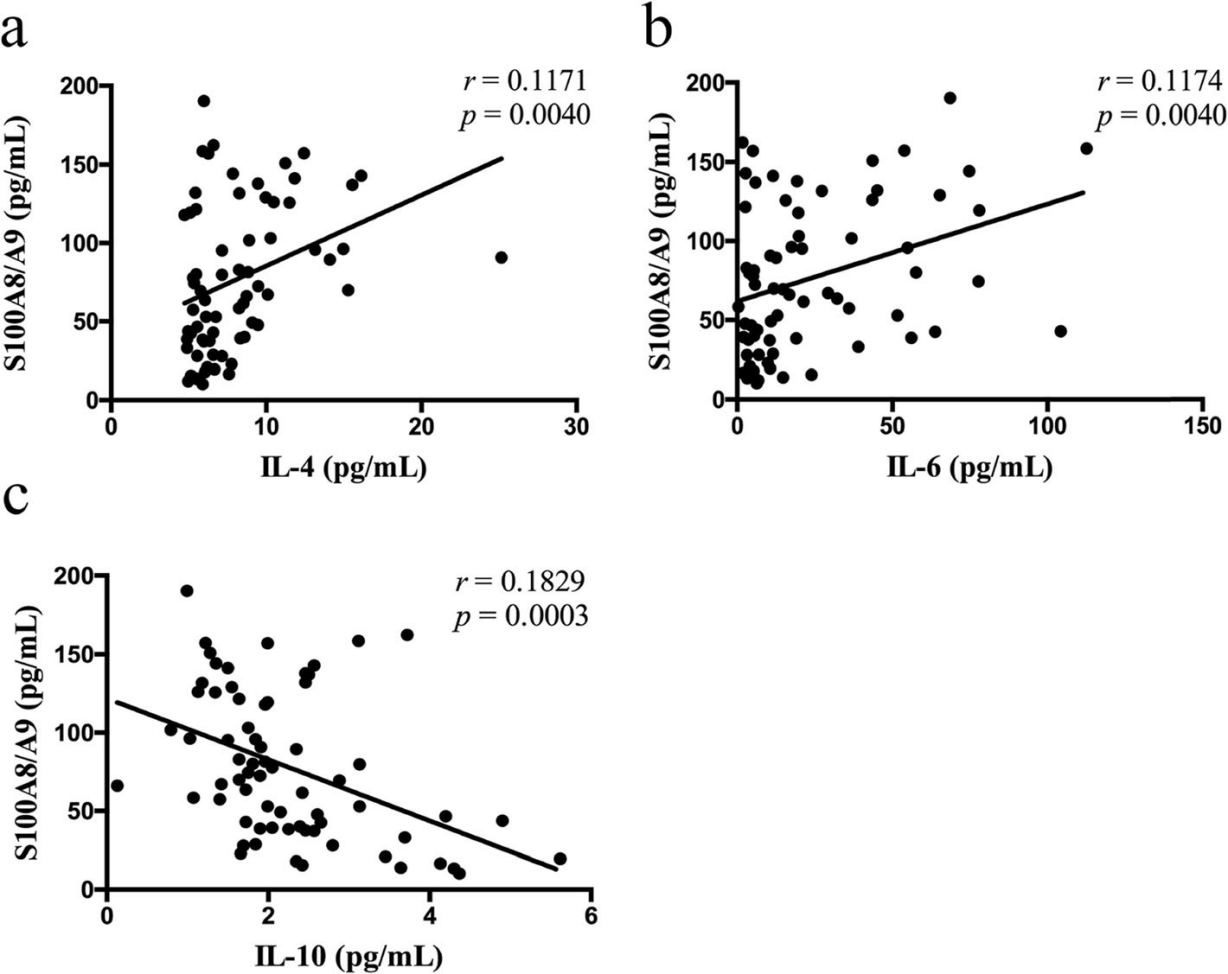
Correlation between IL-4, IL-6, IL-10 and S100A8/A9 levels. The correlation analysis was performed to analyze the concentrations between S100A8/A9 and IL-4 **a**, IL-6 **b** and IL-10 **c** in patients with DM-ILD and controls

We next analyzed the correlation between IL-4, IL-6, IL-10 and S100A8/A9 levels. As shown in Fig. 2, significant positive correlation was found between levels of IL-4, IL-6 and S100A8/A9 (*r*_s_ = 0.1171, *P* = 0.0040; *r*_s_ = 0.1174, *P* = 0.0040). IL-10 levels were significantly negatively correlated with S100A8/A9 levels (*r*_s_ = 0.1829, *P* = 0.0003).

### Correlation between S100A8/A9 levels and disease activity in DM-ILD patients

**Fig. 3:**
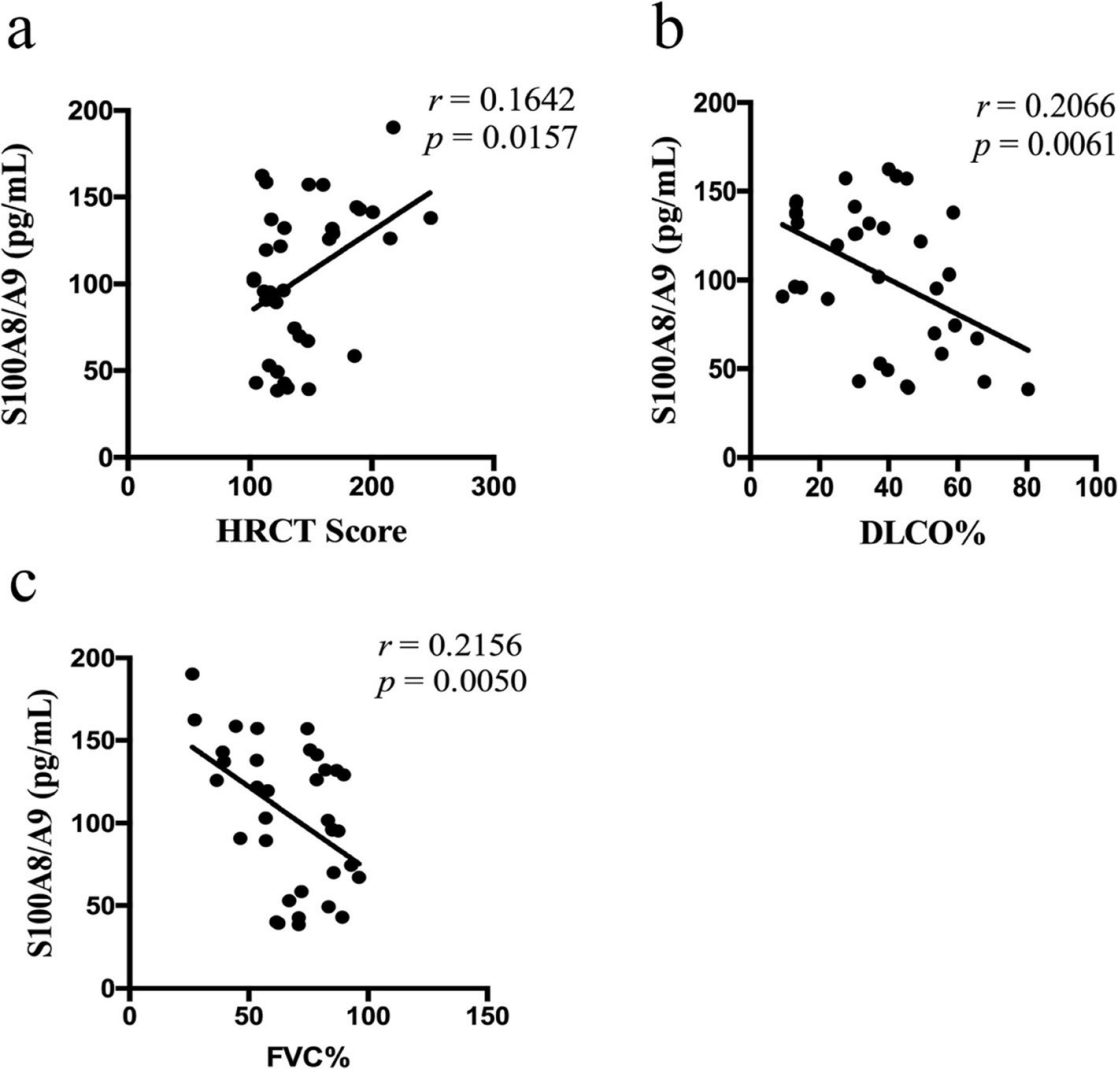
Correlation between S100A8/A9 levels and disease activity in DM-ILD patients. The correlation analysis was performed to analyze the relationship between S100A8/A9 levels and HRCT score **a** and lung functions, including DLCO% **b** and FVC% **c**

To assess the potency of S100A8/A9 as a biomarker to predict the disease activity of DM-ILD, we analyzed the relationship between S100A8/A9 levels and their HRCT score or lung functions in DM-ILD patients. As shown in Fig. 3, the level of S100A8/A9 was significantly positively correlated with HRCT score (r_s_ = 0.1642, *P* = 0.0157) and negatively correlated with DLCO% (*r*_*s*_ = 0.2066, *P* = 0.0061) and FVC% (r_s_ = 0.2156, *P* = 0.0050).

### The risk factor for ILD development in DM patients

The results of univariate and multivariate logistic regression analyses of the risk factors of ILD development in DM patients were shown in Table 3. Univariate logistic regression revealed that ILD development was significantly associated with serum S100A8/A9 levels (OR, 9.600; 95% CI, 1.881–48.999; *p* = 0.007). Serum IL-4, IL-6 levels and DLCO% variables were not significantly associated with ILD development but were included in the multivariable base model (all *p* < 0.2). Multivariate logistic regression demonstrated that only serum S100A8/A9 levels was a significant independent risk factor associated with ILD development (OR, 15.352; 95% CI, 2.411–17.750; *p* = 0.004).

**Table 3:** Logistic regression analysis of risk factors associated with ILD development in DM patients

Parameter	Odds ratio	95% confidence interval	***P*** value
Univariate analysis			
IL-4	1.385	0.093–2.593	0.188
IL-6	2.597	0.628–10.743	0.188
IL-10	1.270	0.374–4.314	0.702
S100A8/A9	9.600	1.881–48.999	0.007
FVC%	0.475	0.125–1.808	0.275
DLCO%	0.389	0.107–1.411	0.151
C-reactive Protein	1.833	0.434–7.736	0.409
Multivariate Analysis			
IL-4	1.199	0.032–2.233	0.083
IL-6	3.448	0.624–19.058	0.156
S100A8/A9	15.352	2.411–17.750	**0.004**
DLCO%	0.352	0.067–1.847	0.217

^a^*CI* confidence interval, *OR* odds ratio

### Correlation between serum S100A8/A9 levels and clinical prognosis

**Fig. 4:**
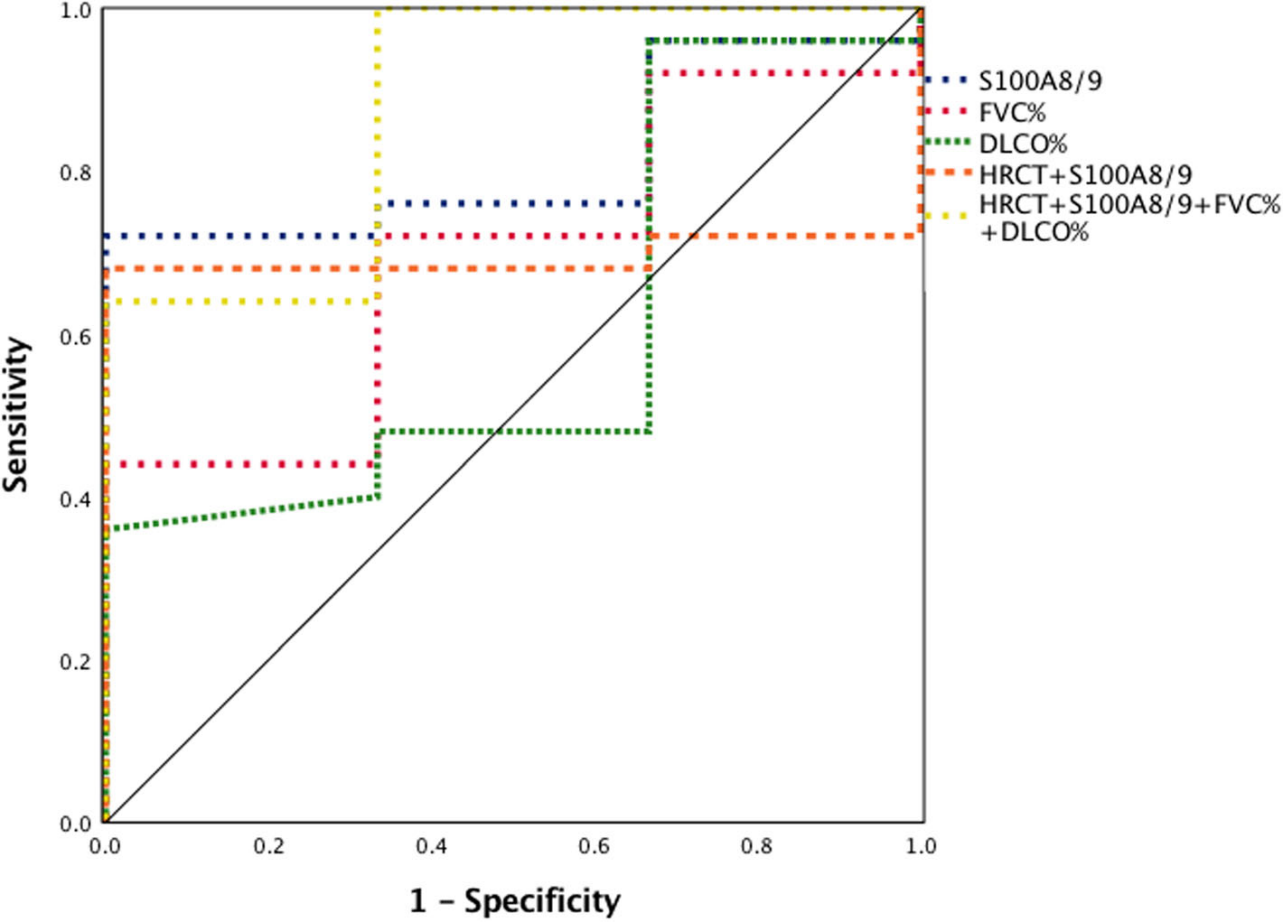
Receiver operating characteristic curve analysis of serum S100A8/A9 levels, pulmonary functions and prognosis of DM patients with ILD. Receiver operating characteristic (ROC) curve analysis of 40 DM patients with ILD according to the serum S100A8/A9 levels, pulmonary functions and combined features to predict the prognosis. The combined feature has better prognostic performance in DM patients with ILD

We evaluated the association among serum S100A8/A9 levels, pulmonary function variables and prognosis of DM patients with ILD ROC curve analysis. As shown in Fig. 4, the area under the curve (AUC) value of S100A8/A9 was 0.81 and that of S100A8/A9-HRCT- DLCO%-FVC% 0.88 (95% CI: 0.636–0.991, *P* = 0.081; 95% CI: 0.672–1.000, *P* = 0.106). Although there was no significant difference between the single and combined features, it suggested that combined features would be more predictive of the clinical outcomes than S100A8/A9 alone.

## Discussion

DM-ILD has high morbidity and mortality. Inflammation plays a key role in the pathogenesis of DM-ILD. S100A8/A9 is mainly released by neutrophils and monocytes, and stable dimers or homodimers can be formed in vitro and in vivo. S100A8/A9 have already been verified to play an important role in the progress of inflammation. Serum S100A8/A9 levels in patients with systemic lupus erythematosus (SLE) are elevated, which may be closely related to disease activity [26, 27].. Elevated S100A9 level in sputum is a potential biomarker of neutrophilic inflammation in severe asthma [28]. Andreasson K et al. found that fecal S100A8/A9 level in patients with systemic sclerosis may be a biomarker of gastrointestinal diseases [29]. In Idiopathic pulmonary fibrosis (IPF), elevated level of S100A9 was observed in bronchoalveolar lavage fluid (BALF) [30, 31]. Hara A et al. reported that S100A9 level in BALF may be a biomarker of IPF fibrosis [32]. Therefore, based on the above researches, we hypothesized that S100A8/A9 may play a role in the development of DM-ILD.

Interleukin-4 (IL-4) is a multifunctional and multipotent cytokine, which plays an important role in proliferation, differentiation and apoptosis of various cell types, mainly secreted by mast cells, Th2 cells, eosinophils and basophils [33]. IL-6 and IL-10 had been confirmed to be associated with A/SIP in patients with DM/CADM [34–36]. IL-6, IL-8, and TNF-α were previously indicated to be associated with overall disease activity in PM/DM [37]. However, the association between pulmonary disease activity and above cytokines has remained unclear.

To our knowledge, this is the first study to show that serum S100A8/A9 levels were significantly enhanced in DM patients with ILD, especially in those with A/SIP, compared with those without ILD. In DM-ILD patients, these concentrations were associated with the disease activity and prognosis. Moreover, serum levels of IL-4, IL-6 were significantly higher in DM-ILD patients than those in healthy controls (*p* = 0.0013, 0.0017). Serum IL-4 (*r* = 0.1171, *p* = 0.0040), IL-6 (*r* = 0.1174, *p* = 0.0040) were significantly correlated with S100A8/A9 in patients with DM-ILD. Our findings were consistent with the previous studies that S100A8/A9, IL-4, IL-6 levels were increased in inflammatory diseases, and they may play a key role in the DM-ILD.

Different from previous studies, we found that serum IL-10 levels of DM-ILD patients were dramatically lower than controls (*p* = 0.0001). It has been indicated that IL-10 family is comprised of nine members which are powerful immune mediators with versatile functions, including reducing tissue damage caused by excess and uncontrolled inflammatory effector responses [38]. Several recent studies showed that the transcription factor c-Maf up-regulated IL-10 production in vivo in CD4+ T cells from Th1, Th2, and Th17 cells in experimental disease models and Bhlhe40 repressed IL-10 production in T cells during immune responses [39, 40]. Thus, we hypothesize that the development of DM-ILD may be associated with the absence of c-Maf or the overexpression of Bhlhe40 in CD4+ T cells. But it remains to be confirmed in the further research.

ILD is common among patients with DM, and is often associated with a worse prognosis [41]. HRCT scoring system can be used as a parameter to evaluate the damage of ILD structure, and predict the severity and prognosis of DM-ILD [42]. Furthermore, in patients with IPF, forced vital capacity (FVC) and carbon monoxide diffusion (DLCO) are considered to be the most sensitive parameters for evaluating the course of the disease [43].. Hence, here we used FVC% and DLCO% as pulmonary function impairment evaluation indicators, combined with HRCT score system, to predict the severity and prognosis of DM-ILD.

We found that serum levels of S100A8/A9 were significantly correlated with ILD structural damage (HRCT score) (*r* = 0.1642, *p* = 0.0157) and pulmonary function impairment (DLCO%: *r* = − 0.2066, *p* = 0.0061, FVC%: *r* = − 0.2156, *p* = 0.0050). The correlation between serum S100A8/A9 levels and HRCT and PFT impairment suggested that high serum levels of S100A8/A9 directly reflected ILD severity in patients with DM-ILD.

There are several limitations to this study. Firstly, this study was retrospectively conducted. Secondly, analysis of DM-ILD patients with anti-MDA5 antibodies was not performed. Thirdly, some patients were being treated with prednisolone at the time of serum collection. These medications maybe influenced the measurement of cytokine levels. Further studies are required to investigate the precise mechanisms by which S100A8/A9 contributes to DM-ILD.

## Conclusions

In conclusion, this study demonstrated that serum S100A8/A9 levels were elevated in DM patients with ILD, in particular those with A/SIP. These levels were highly correlated with IL-4, IL-6 and IL-10 levels and were significantly correlated with HRCT score, FVC% and DLCO%. These cytokines may contribute to the pathogenesis of DM-ILD. Our results suggest that S100A8/A9 may be a useful biomarker for assessing ILD activity and predicting prognosis in DM patients with ILD.

## Data Availability

The dataset used and/or analyzed during the current study are available from the corresponding author on reasonable request.

## Abbreviations

A/SIP: Acute/subacute interstitial pneumonia
BALF: Bronchoalveolar lavage fluid
CADM: Clinically amyopathic dermatomyositis
CBA: Cytometric beads array
CIP: Chronic interstitial pneumonia
CK: Creatine kinase
CRP: C-reactive protein
DLCO%: % Diffusing capacity of the lungs for carbon monoxide
DM: Dermatomyositis
ELISA: Enzyme-linked immunosorbent assay
ESR: Erythrocyte sedimentation rate
FVC%: % Forced vital capacity
GGO: Ground glass opacities
HRCT: High-resolution computed tomography
IFN: Interferon
IIM: Idiopathic inflammatory myopathy
IL: Interleukin
ILD: Interstitial lung disease
IPF: Idiopathic pulmonary fibrosis
Th: T-helper
TLR: Toll-like receptor
TNF- α: Tumor necrosis factor- α
SLE: Systemic lupus erythematosus
